# Gallium-68 (68Ga)-Labelled Prostate-Specific Membrane Antigen Positron Emission Tomography/Computed Tomography in the Initial Staging of High-Risk Prostatic Adenocarcinoma: Reclassification to Oligometastatic Disease and Change in Treatment Intent

**DOI:** 10.7759/cureus.116379

**Published:** 2026-09-16

**Authors:** Carlos Paul Villalba-Meneses, Adriana P Noboa, Veronica Marcela Villegas-Paredes

**Affiliations:** 1 Nuclear Medicine Department, Universidad Indoamérica, Quito, ECU; 2 PET Scan Unit, Hospital Metropolitano, Quito, ECU; 3 Internal Medicine Department, Universidad de Guayaquil, Guayaquil, ECU

**Keywords:** 68ga-psma-i&t, neoplasm metastasis, neoplasm staging, pet/ct scan, prostatic neoplasms

## Abstract

Prostate cancer is among the most frequently diagnosed malignancies worldwide. In high-risk disease, conventional imaging underestimates nodal and skeletal extent, so treatment intent may be defined on incomplete staging. Positron emission tomography (PET) with gallium-68 (68Ga)-labelled prostate-specific membrane antigen (PSMA) ligands has shown superior diagnostic accuracy in this setting.

A 69-year-old man, Eastern Cooperative Oncology Group (ECOG) performance status 0, presented with urinary symptoms and a rising prostate-specific antigen, from 18.2 to 34.1 ng/mL, with a doubling time under three months. Multiparametric magnetic resonance imaging (MRI) showed a Prostate Imaging-Reporting and Data System (PI-RADS) 5 lesion with extraprostatic extension, and biopsy of 12 cores (six per lobe) demonstrated acinar adenocarcinoma, Gleason 8 (4+4), International Society of Urological Pathology (ISUP) grade group 4, with perineural invasion and involvement of five of six left-sided cores; all six right-sided cores were tumor-free. PET/computed tomography (CT) was performed on a digital Siemens Biograph Vision 450 (Erlangen, Germany) after intravenous administration of 110.63 MBq (2.99 mCi) of 68Ga-PSMA imaging and therapy (I&T), with acquisition at 70 minutes and a delayed thoracic study. Images were read using the Prostate Cancer Molecular Imaging Standardized Evaluation (PROMISE) V2 criteria with molecular imaging Tumor, Node, Metastasis (miTNM) staging.

With background references of standardized uptake value normalized to lean body mass (SUL) 1.2 for blood pool, 4.0 for liver, and 20.1 for parotid gland, the left-lobe prostatic lesion showed SUL 10.6, PSMA-expressing tumor volume (PSMA-TV) 10.09 cc, and PSMA total lesion (PSMA-TL) 53.23, with a PRIMARY score of 4. A 15 mm left internal iliac node with SUL 6.9, an 8 mm para-aortic node with SUL 3.2, and a 6 mm blastic lesion in the right sixth anterior costal arch with SUL 1.4, rising to 1.5 on delayed imaging, were identified. Whole-body tumor burden was PSMA-TV 15.09 cc and PSMA-TL 60.9. Staging was miT3a, miN1, miM1a, and unifocal miM1b, the latter based on an indeterminate bone lesion. The original treatment plan, prostate and pelvic radiotherapy with short-course androgen blockade and locoregional curative intent, was replaced by androgen deprivation with enzalutamide, prostate and pelvic radiotherapy, and stereotactic body radiotherapy to the para-aortic node and the costal lesion. Prostate-specific antigen fell to 0.15 ng/mL at three months and to undetectable levels at the six-month assessment.

Molecular imaging reclassified disease regarded as high-risk localized as presumed low-volume oligometastatic and changed treatment intent from locoregional curative to intensified systemic therapy with metastasis-directed radiotherapy, with an early deep biochemical response on follow-up, which does not by itself establish durable oncological control.

## Introduction

Prostate cancer is the fourth most common malignancy worldwide, with an age-standardized incidence rate of 29.4 per 100,000 men [1]. Incidence and mortality are particularly high in Latin America and the Caribbean, including Ecuador, where a large proportion of cases present at an advanced stage [2]. Risk stratification relies on prostate-specific antigen (PSA), clinical stage, and Gleason grading, reorganized by the International Society of Urological Pathology (ISUP) into grade groups 1 through 5 [3]; a Gleason score of 8 (4+4, grade group 4) together with a PSA above 20 ng/mL defines a high-risk scenario. Multiparametric magnetic resonance imaging (MRI) interpreted with the Prostate Imaging-Reporting and Data System (PI-RADS) guides biopsy [4], but its performance for nodal and skeletal staging is limited, and conventional imaging (computed tomography (CT) and bone scintigraphy) has insufficient sensitivity for early metastatic disease.

Prostate-specific membrane antigen (PSMA) is a transmembrane glycoprotein overexpressed on prostatic adenocarcinoma cells in proportion to tumor grade, and gallium-68 (68Ga)-labelled ligands directed against it enable whole-body imaging with a high lesion-to-background ratio, as detailed in the current joint European Association of Nuclear Medicine (EANM)/Society of Nuclear Medicine and Molecular Imaging (SNMMI) procedure guideline [5]. In the randomized proPSMA trial, 68Ga-PSMA-11 positron emission tomography (PET)/CT achieved 92% accuracy versus 65% for conventional imaging in detecting nodal and metastatic disease, with a markedly greater impact on treatment decisions [6]; prespecified follow-up confirmed that PET-defined nodal status is independently prognostic [7]; and a recent meta-analysis has consolidated this diagnostic performance [8].

Reproducible interpretation depends on structured reporting frameworks: the Prostate Cancer Molecular Imaging Standardized Evaluation (PROMISE) molecular imaging Tumor, Node, Metastasis (miTNM) classification [9], later refined as PROMISE V2 with a four-grade PSMA expression scale [10], grades tracer uptake in the primary tumor and each nodal or distant lesion from 0 (no uptake) to 3 (uptake exceeding the liver), analogous to the anatomical grading used in conventional TNM staging. The PSMA Reporting and Data System (PSMA-RADS) [11] similarly assigns a five-point likelihood-of-malignancy score to individual lesions; the E-PSMA guidelines [12] standardize acquisition and reporting; and the PRIMARY score [13] applies a comparable five-point scale to intraprostatic uptake patterns on PET alone. These frameworks exist because PSMA expression is not exclusive to prostatic tumor tissue: benign processes such as healing rib fractures, fibrous dysplasia, and degenerative or inflammatory bone changes can also express PSMA on their associated vasculature, producing low-intensity uptake indistinguishable from early metastatic disease on visual assessment alone. This non-specific bone uptake, particularly in solitary rib lesions, is a well-recognized pitfall that can shift a patient from localized or nodal disease to metastatic bone disease and change treatment intent [14].

We report a case in which 68Ga-PSMA imaging and therapy (I&T) PET/CT combined both aspects of this technology: it provided nodal and skeletal information unavailable by other means and produced a documented change in management while also requiring a judgement call on a single bone lesion with low-grade expression at the limit of the technique's specificity. We describe these findings, critically analyze the miTNM category assignments, and document their impact on treatment and outcome.

## Case presentation

A 69-year-old man, Eastern Cooperative Oncology Group (ECOG) performance status 0 [15], presented with urinary complaints and elevated PSA. He had no prior oncological treatment, no relevant family history of prostate, breast, ovarian, or pancreatic cancer, and no smoking history. Digital rectal examination revealed a prostate with stony-hard consistency in the left lobe (International Prostate Symptom Score (IPSS): 18 [16]). His initial PSA was 18.2 ng/mL, rising to 25.9 ng/mL one month later and to 34.1 ng/mL at three months, with a doubling time under three months; baseline testosterone was 450 ng/dL, confirming a hormone-sensitive state.

Multiparametric MRI showed a 49 g prostate with a poorly defined lesion in the anterior and left posterior peripheral zone extending from mid gland to base, with extraprostatic extension and no adequate capsular cleavage plane (PI-RADS 5; Figure 1). Biopsy, comprising 12 cores (six per lobe), confirmed acinar adenocarcinoma, Gleason 8 (4+4), ISUP grade group 4, with five of six left-sided cores positive (70-90% involvement) and perineural invasion; all six right-sided cores were tumor-free. Given the ISUP grade group 4, rapidly rising PSA, and extraprostatic extension, 68Ga-PSMA-I&T PET/CT was requested for staging before starting the originally planned locoregional radiotherapy with short-course androgen blockade. 68Ga-PSMA-I&T PET/CT was performed as the sole upfront staging modality beyond multiparametric MRI; conventional staging with whole-body bone scintigraphy and contrast-enhanced CT of the chest, abdomen, and pelvis was not performed.

**Figure 1:**
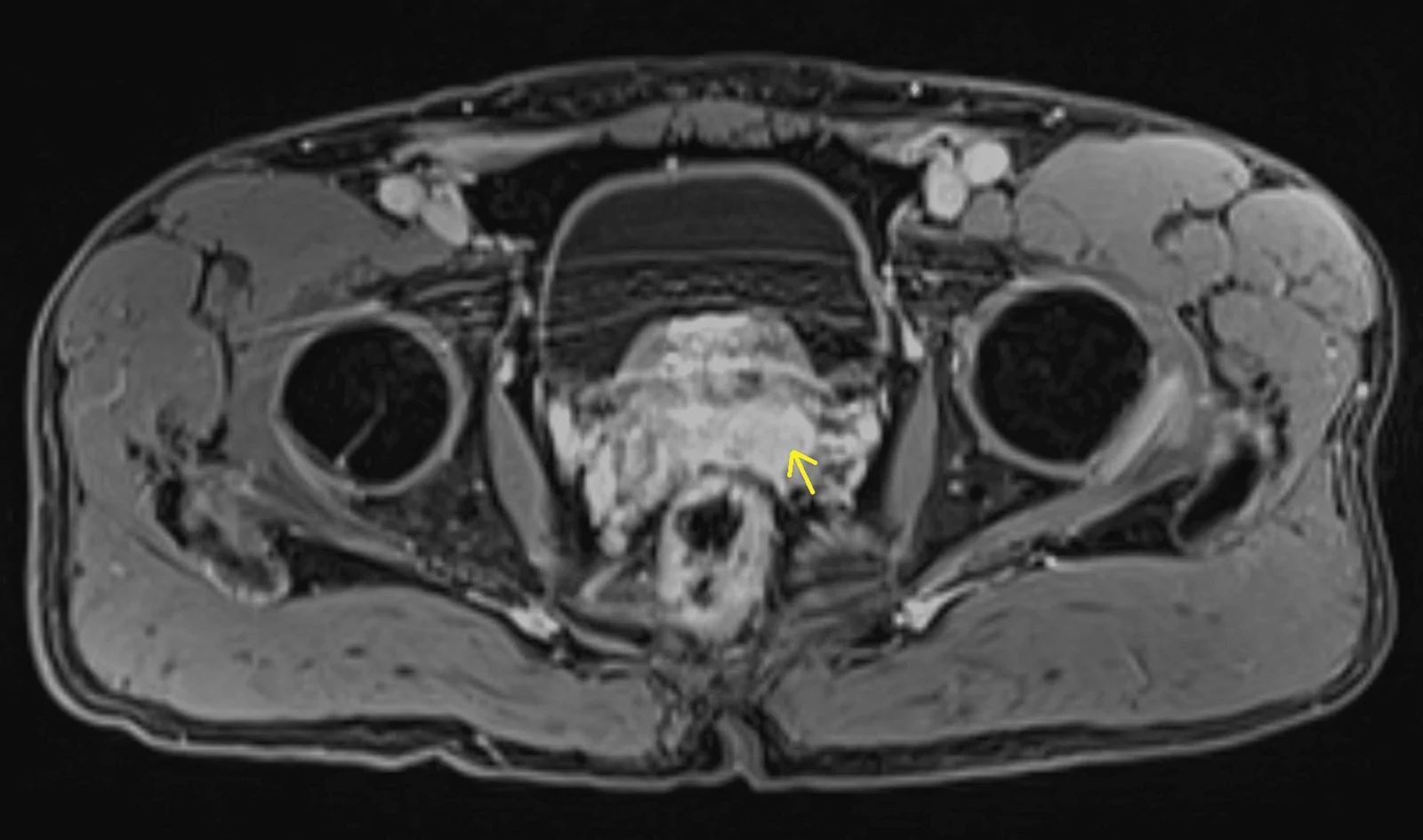
Contrast-enhanced multiparametric magnetic resonance imaging (mpMRI) of the prostate Yellow arrow: primary prostatic lesion in the left posterolateral peripheral zone, showing enhancement on a contrast-enhanced T1-weighted sequence and loss of the capsular plane consistent with extraprostatic extension (PI-RADS 5).

PET/CT was performed on a digital scanner (Siemens Biograph Vision 450, Erlangen, Germany) 70 minutes after intravenous administration of 110.63 MBq (2.99 mCi) of 68Ga-PSMA-I&T, with dynamic prostatic imaging, whole-body acquisition, and a delayed thoracic series. Background standardized uptake value normalized to lean body mass (SUL) values (blood pool 1.2, liver 4.0, parotid 20.1) confirmed preserved biodistribution (Figure 2).

**Figure 2:**
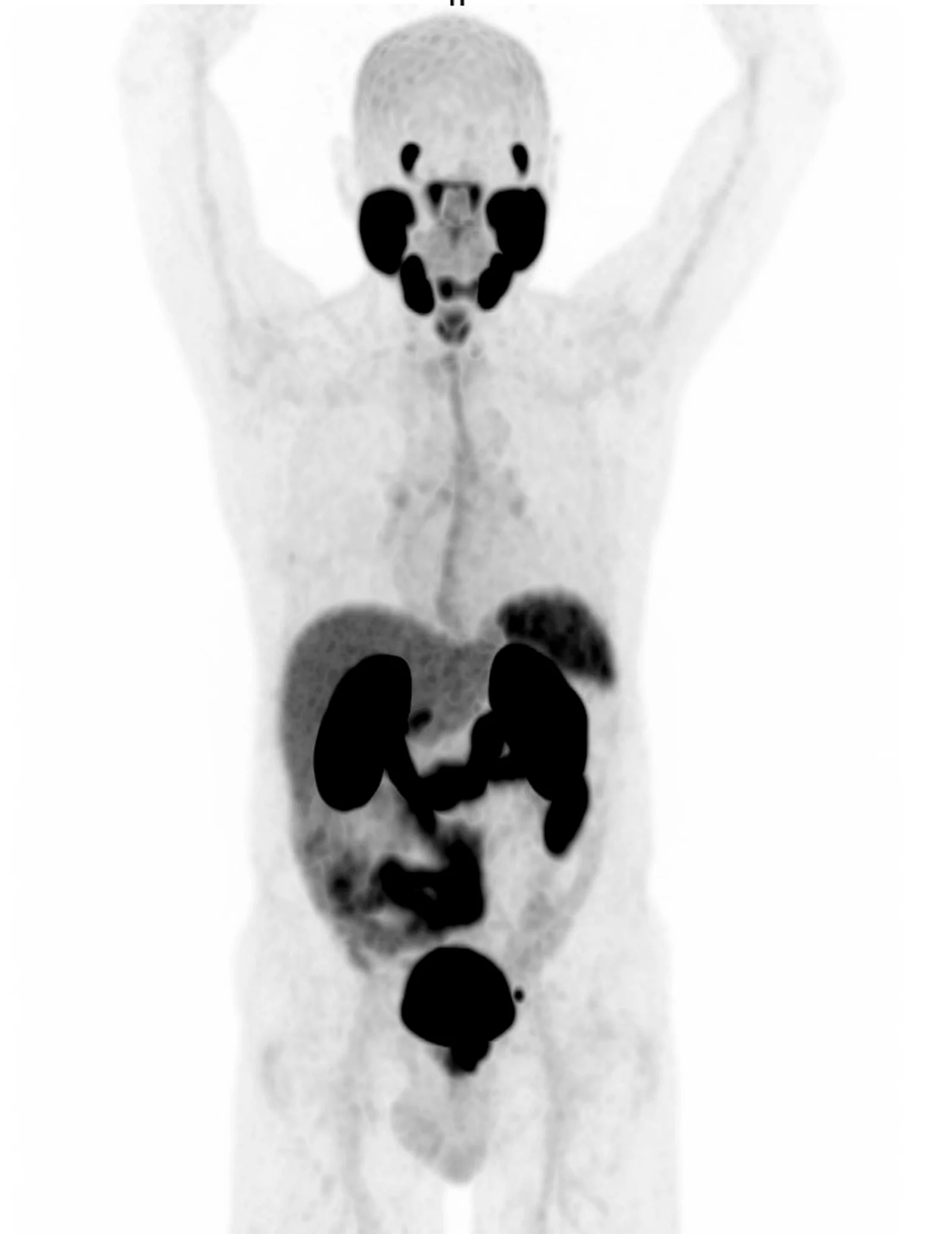
Radiopharmaceutical biodistribution

The primary tumor showed intense uptake in the left lobe (SUL 10.6, PRIMARY score 4) on axial CT (Figure 3A), fused PET/CT (Figure 3B), and PET-only axial (Figure 3C) and coronal (Figure 3D) images, concordant with the PI-RADS 5 lesion and biopsy laterality.

**Figure 3:**
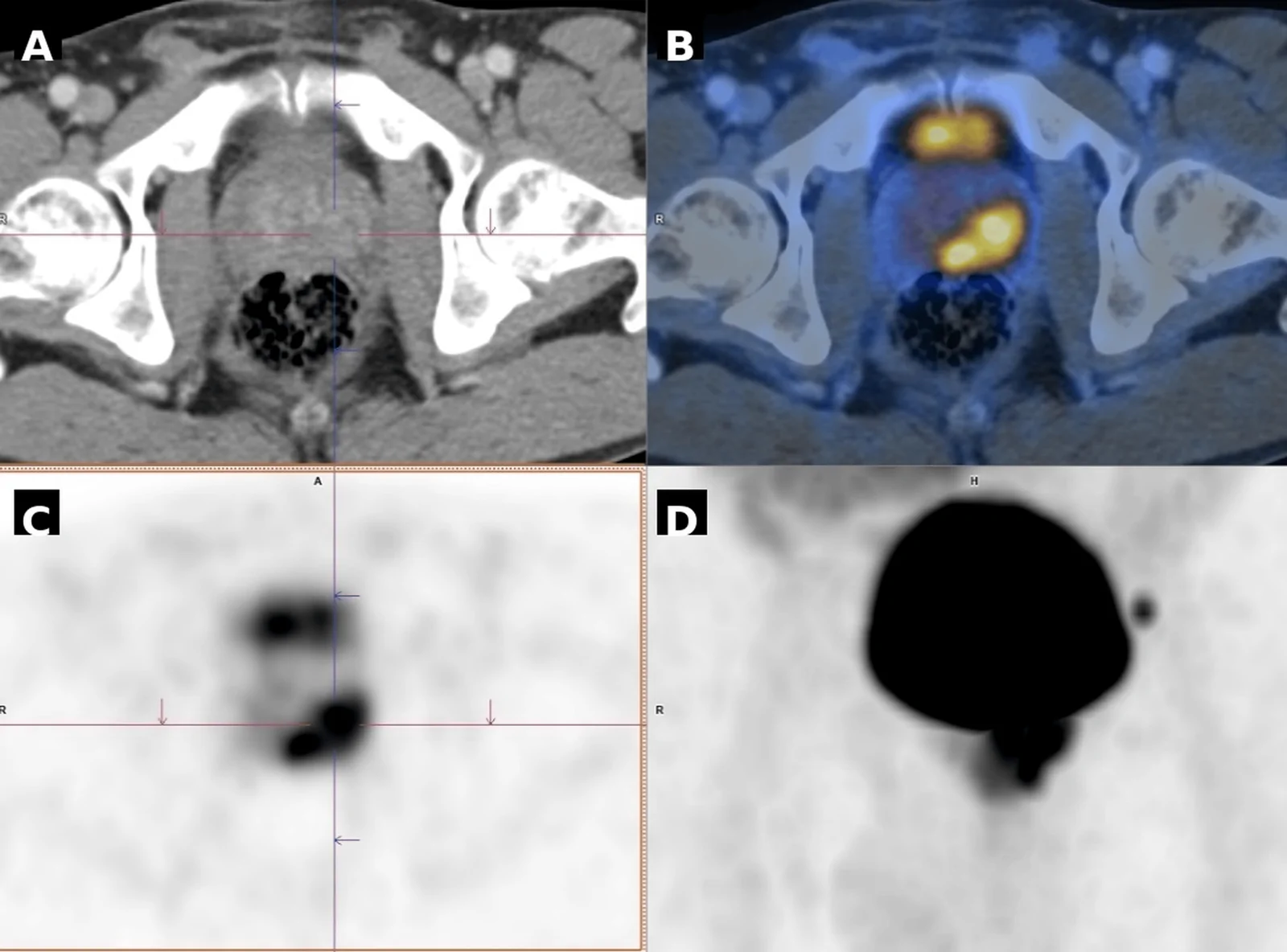
Gallium-68 (68Ga)-labelled prostate-specific membrane antigen (PSMA) positron emission tomography (PET)/computed tomography (CT) study depicting a prostatic lesion

A 15 mm left internal iliac node (SUL 6.9) was identified in the regional drainage territory on axial CT (Figure 4A), fused PET/CT (Figure 4B), and PET-only axial (Figure 4C) and coronal (Figure 4D) images, together with an 8 mm para-aortic node (SUL 3.2) in extraregional territory on axial CT (Figure 5A), fused PET/CT (Figure 5B), and PET-only axial (Figure 5C) and coronal (Figure 5D) images.

**Figure 4:**
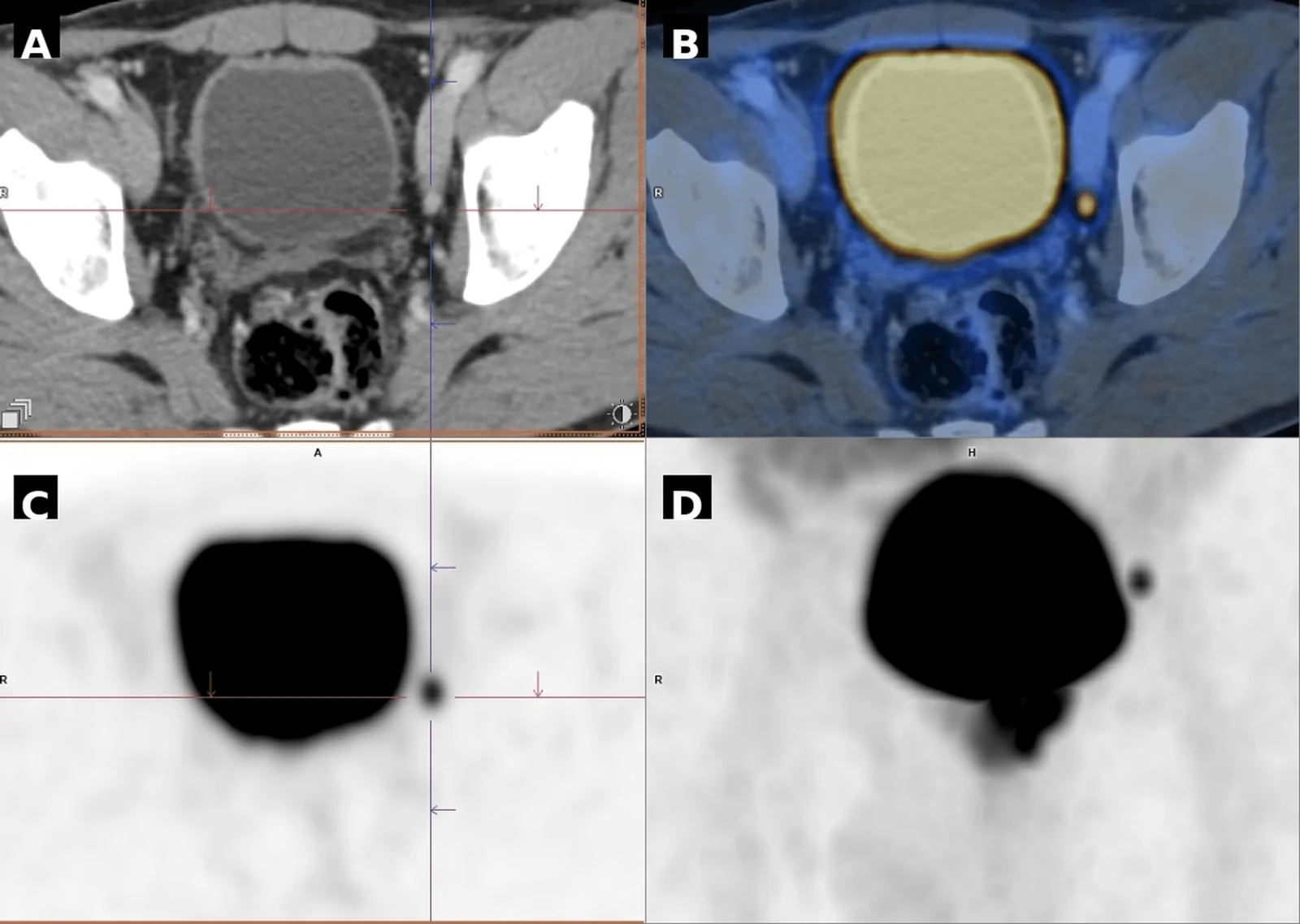
Gallium-68 (68Ga)-labelled prostate-specific membrane antigen (PSMA) positron emission tomography (PET)/computed tomography (CT) study depicting pelvic lymphadenopathy

**Figure 5:**
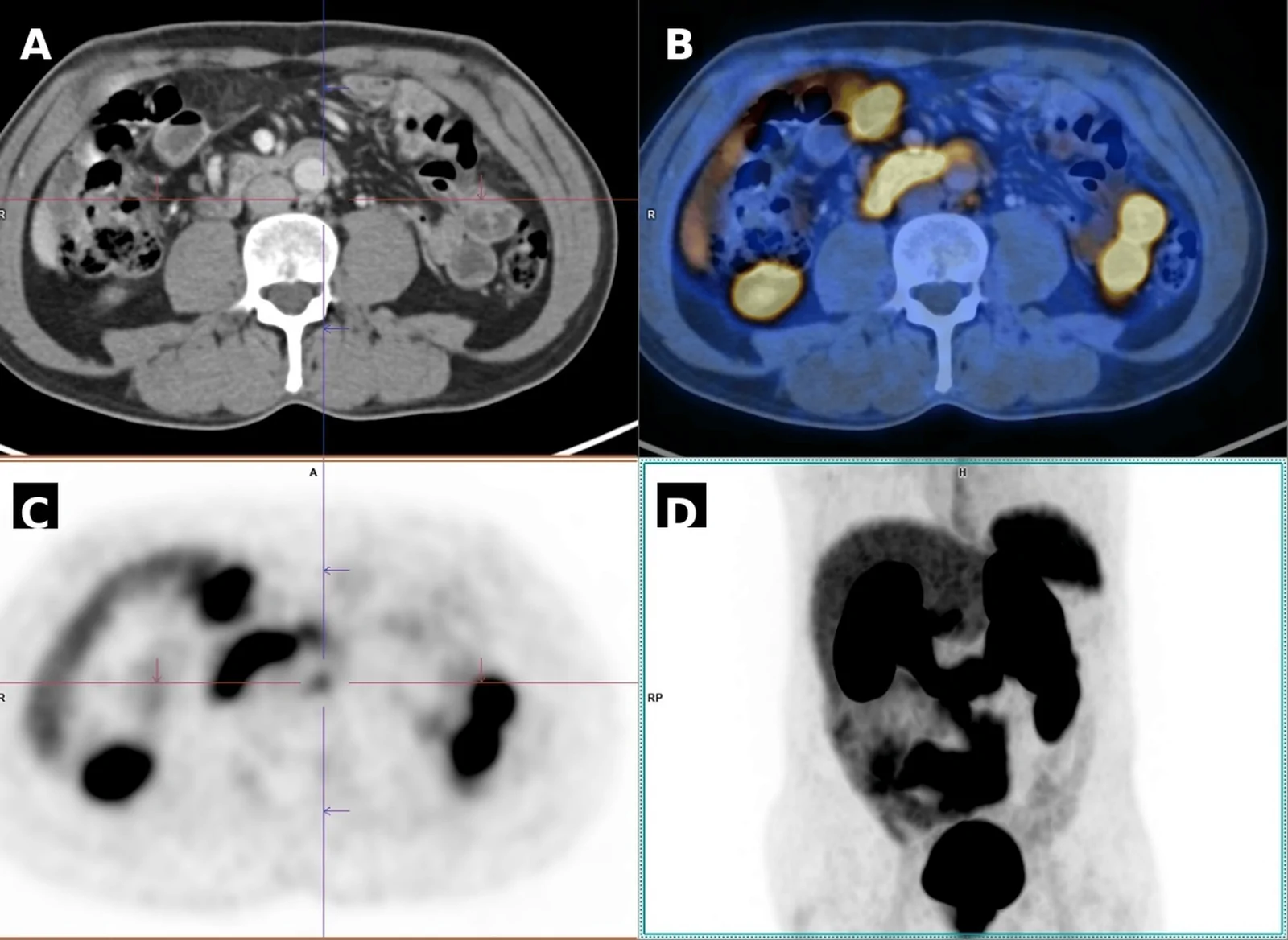
Gallium-68 (68Ga)-labelled prostate-specific membrane antigen (PSMA) positron emission tomography (PET)/computed tomography (CT) study depicting retroperitoneal lymphadenopathy

A 6 mm blastic lesion in the right sixth anterior costal arch, seen on CT (Figure 6A) and fused PET/CT (Figure 6B), showed low-grade uptake on PET-only images (Figure 6C-6D) (SUL 1.4, rising to 1.5 on delayed imaging).

**Figure 6:**
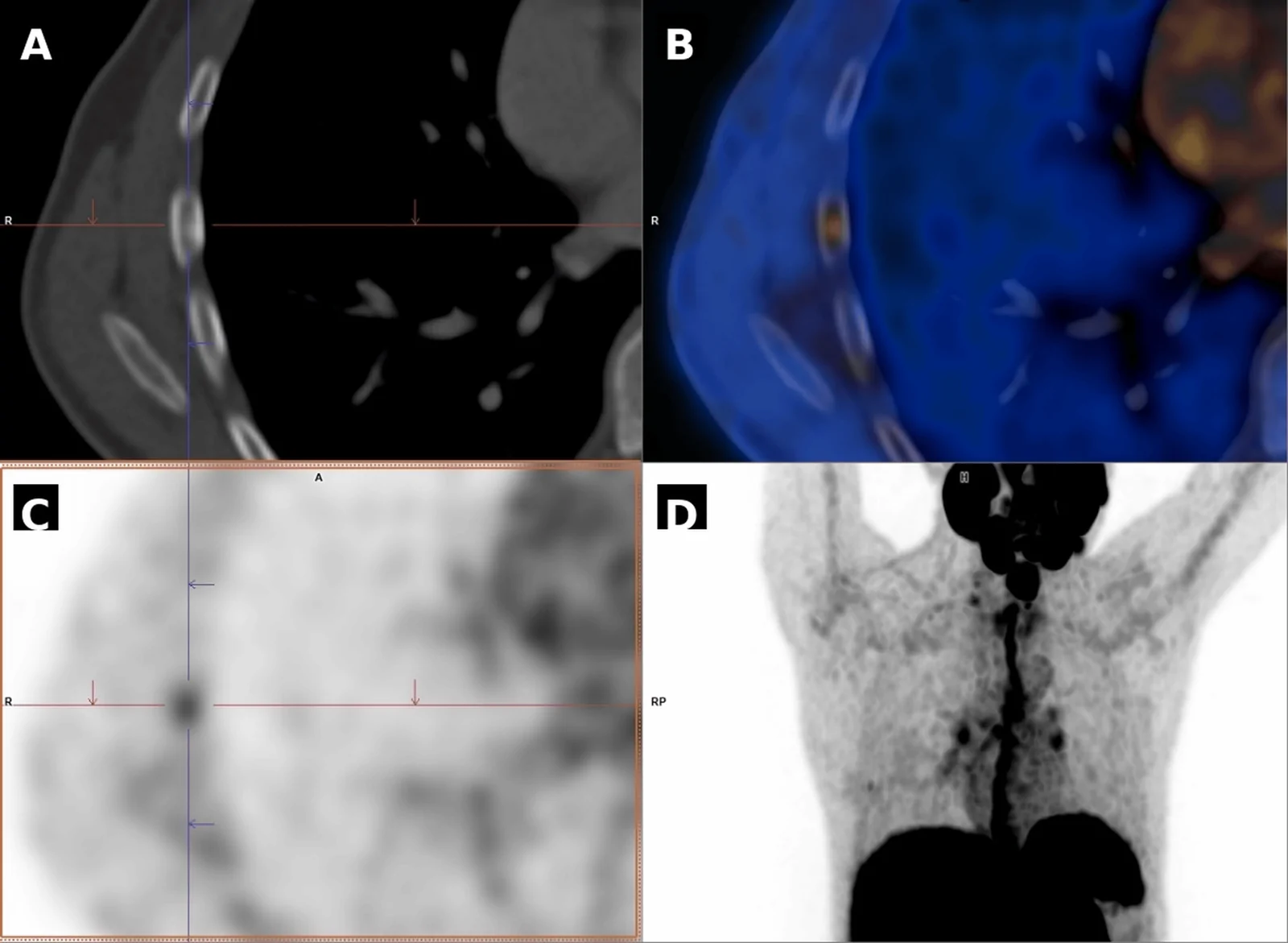
Gallium-68 (68Ga)-labelled prostate-specific membrane antigen (PSMA) positron emission tomography (PET)/computed tomography (CT) study depicting a costal bone lesion

No other suspicious nodal, visceral, or osseous lesions were seen; two benign incidental findings (a hepatic cyst and splenic granulomas) showed no tracer uptake. Whole-body tumor burden was PSMA-expressing tumor volume (PSMA-TV) 15.09 cc and PSMA total lesion (PSMA-TL) 60.9.

Applying the PROMISE V2 criteria, the case was staged miT3a, miN1, miM1a, and unifocal miM1b (cT3a cN1 M1b, stage IVB), the latter based on an indeterminate bone lesion discussed further below. The primary tumor and the iliac node were confirmed with high certainty across modalities. The para-aortic node, despite its subcentimeter size, was interpreted as involved based on grade 1 PSMA expression, precisely the scenario in which molecular imaging adds the most over anatomical criteria. The rib lesion was the most uncertain finding: its blastic morphology, low but rising uptake, and the context of high-grade disease with two-territory nodal involvement supported a metastatic interpretation, though non-specific bone uptake and a solitary rib location are recognized pitfalls of PSMA PET/CT [14]. No lesion was histologically confirmed, the main limitation of the case.

The findings changed management: the plan shifted from locoregional radiotherapy with short-course androgen blockade to intensified systemic therapy (androgen deprivation plus an androgen receptor pathway inhibitor) combined with the radical treatment of the primary and pelvis and metastasis-directed stereotactic radiotherapy to the para-aortic node and rib lesion. Notably, the para-aortic node alone was sufficient to justify systemic intensification; the rib lesion, despite its greater diagnostic uncertainty, only added a target for ablative radiotherapy and did not independently change the systemic strategy.

The patient received leuprolide acetate, enzalutamide, and calcium/cholecalciferol supplementation, together with volumetric modulated arc radiotherapy to the prostate and pelvis and stereotactic body radiotherapy to the para-aortic node and rib lesion. At three months, PSA fell to 0.15 ng/mL with testosterone in the castrate range; at six months, PSA was undetectable (<0.05 ng/mL), and follow-up CT showed sclerotic healing of the rib lesion, consistent with treatment response.

## Discussion

This case illustrates why PSMA-targeted imaging has become the reference standard for the initial staging of high-risk prostate cancer: a single study moved a patient from being a candidate for locoregional treatment with curative intent to being classified as low-volume oligometastatic disease, with a complete reformulation of the treatment plan. Beyond this, the case shows something useful for clinical decision-making: the finding that actually changed treatment (the para-aortic node) was also the most certain one, while the least certain finding (the rib lesion) had the least impact on treatment. This will not always be the case, so it is worth pointing out. This magnitude of effect matches the evidence base: in the proPSMA trial, 68Ga-PSMA-11 PET/CT achieved 92% accuracy versus 65% for conventional imaging, with a markedly greater impact on management [6]; PET-defined nodal status carries independent prognostic value [7]; and the most recent meta-analysis has consolidated this performance for primary staging [8].

The intensity of uptake in the primary lesion, grade 2 molecular imaging PSMA (miPSMA) expression and a PRIMARY score of 4 [10,13], is consistent with PSMA expression scaling with tumor grade in this ISUP grade group 4 adenocarcinoma.

The case also illustrates the specific contribution of molecular over anatomical imaging. The internal iliac node showed concordant morphological (15 mm) and functional (grade 2) criteria, but the para-aortic node, only 8 mm, below the conventional size threshold, was interpreted as involved based on grade 1 expression alone. It was this subcentimeter node, not the bone lesion, that determined the change in treatment intent, precisely the scenario in which molecular imaging adds the most value.

The rib lesion remains the point of greatest interpretive interest. Non-specific bone uptake is a well-characterized pitfall of PSMA PET/CT: a systematic review of PSMA-avid rib lesions found that most solitary lesions are benign, particularly when uptake is low and no suspicious lesions coexist elsewhere, and warned that overinterpretation shifts patients toward higher metastatic volume with corresponding therapeutic consequences [14]. This patient's lesion met several of those benignity descriptors and was nonetheless treated as metastatic, based on its blastic morphology on contrast-enhanced CT, an increase in uptake on delayed imaging (1.4-1.5), and a high-grade disease context with nodal involvement in two territories; thoracic trauma was excluded on history. Subsequent sclerotic healing on follow-up CT retrospectively supports this reading, though post-radiotherapy sclerosis is not specific to the tissue's prior nature and the lesion was never biopsied.

The most instructive aspect of the case is that this uncertainty was clinically tolerable: the para-aortic node had already established the metastatic scenario, so the finding with the lowest diagnostic certainty was also the one with the lowest therapeutic consequence. When a single low-expression bone lesion is instead the only distant finding, the element that alone decides between curative and systemic intent, the bar for confirmation should be considerably higher.

This case also illustrates PSMA-driven stage migration: patients once treated as localized high-risk disease are now reclassified as metastatic, which can inflate the apparent outcomes of both groups without any real change in biology. Because conventional staging was not performed in this patient, the reclassification described here reflects a comparison against localized disease as assumed from multiparametric MRI and clinical risk factors alone, rather than a documented upgrade from a negative conventional bone scan and CT; this distinction is relevant when interpreting the magnitude of stage migration attributed to PSMA PET/CT in this case. This calls for caution when comparing PSMA-staged outcomes with historical series, including the early biochemical response observed here, which reflects a limited follow-up under active androgen-receptor-axis blockade.

The management adopted is consistent with the available evidence for low-volume disease (a single bone deposit, no visceral disease) per the CHAARTED (ChemoHormonal therapy versus Androgen ablation Randomized Trial for Extensive Disease in prostate cancer) definition [17]: radiotherapy to the primary improves survival in this subgroup per STAMPEDE (Systemic Therapy in Advancing or Metastatic Prostate cancer: Evaluation of Drug Efficacy) [18], enzalutamide with androgen deprivation is supported by ENZAMET (Enzalutamide in first-line androgen deprivation therapy for Metastatic prostate cancer; official title: "Randomised Phase 3 Trial of Enzalutamide in First Line Androgen Deprivation Therapy for Metastatic Prostate Cancer") [19], and metastasis-directed therapy is supported by the ORIOLE (Observation versus stereotactic ablative RadiatIon for OLigometastatic prostate CancEr) trial [20], though ORIOLE was conducted in oligorecurrent disease without concurrent hormonal treatment and with oligometastases defined on conventional imaging, so its extrapolation to a synchronous, PSMA-staged, hormone-sensitive presentation is not direct.

Finally, this case underscores the value of applying structured reporting frameworks effectively rather than declaratively [11], particularly in a healthcare context such as Ecuador's, where access to molecular imaging is expanding rapidly.

Limitations

The following are the limitations of the case: (1) absence of histological confirmation of the nodal and bone lesions, which precludes a reference standard; (2) absence of conventional staging (bone scintigraphy and contrast-enhanced CT) prior to PSMA PET/CT, meaning stage migration is described relative to clinical and multiparametric MRI-based risk assessment rather than a documented conventional imaging baseline; (3) absence of a follow-up PET/CT study that would allow formal molecular response criteria to be applied; and (4) follow-up still short for assessing oncological outcomes beyond the biochemical response.

## Conclusions

In this patient with high-risk prostatic adenocarcinoma, 68Ga-PSMA-I&T PET/CT reclassified disease that conventional staging had considered localized as presumed low-volume oligometastatic (based on imaging criteria without histological confirmation of the nodal or bone lesions), prompting a shift from locoregional treatment with curative intent to an intensified systemic strategy combined with metastasis-directed therapy.

The finding that actually drove this change was a small, non-enlarged para-aortic lymph node that anatomical imaging would not have flagged as abnormal, a clear illustration of what molecular imaging adds beyond morphology alone. A single low-grade bone lesion, in contrast, carried far less diagnostic certainty but also far less therapeutic weight, since the systemic strategy had already been established by the nodal finding; this case suggests that the threshold for confirming an indeterminate lesion should depend on how much that lesion alone would change management, rather than on its probability of representing disease alone.

Complete technical documentation and the effective use of structured reporting systems made the study reproducible and helped identify and correct inconsistencies in the initial interpretation. The undetectable PSA at six months is an expected pharmacological and radiotherapeutic response while androgen-receptor-axis blockade remains active, and it does not by itself establish durable oncological control from the metastasis-directed strategy. Longer follow-up, including castration-resistant progression-free survival, will be required before conclusions can be drawn about the long-term efficacy of the multimodal approach; the biochemical response reported here should therefore be interpreted as an early treatment-response marker rather than evidence of sustained metastatic control.

## References

[1] Bray F, Laversanne M, Sung H, Ferlay J, Siegel RL, Soerjomataram I, Jemal A (2024). Global cancer statistics 2022: GLOBOCAN estimates of incidence and mortality worldwide for 36 cancers in 185 countries. CA Cancer J Clin.

[2] Schafer EJ, Laversanne M, Sung H (2025). Recent patterns and trends in global prostate cancer incidence and mortality: an update. Eur Urol.

[3] Epstein JI, Egevad L, Amin MB, Delahunt B, Srigley JR, Humphrey PA (2016). The 2014 International Society of Urological Pathology (ISUP) consensus conference on Gleason grading of prostatic carcinoma: definition of grading patterns and proposal for a new grading system. Am J Surg Pathol.

[4] Weinreb JC, Barentsz JO, Choyke PL (2016). PI-RADS Prostate Imaging - Reporting and Data System: 2015, version 2. Eur Urol.

[5] Fendler WP, Eiber M, Beheshti M (2023). PSMA PET/CT: joint EANM procedure guideline/SNMMI procedure standard for prostate cancer imaging 2.0. Eur J Nucl Med Mol Imaging.

[6] Hofman MS, Lawrentschuk N, Francis RJ (2020). Prostate-specific membrane antigen PET-CT in patients with high-risk prostate cancer before curative-intent surgery or radiotherapy (proPSMA): a prospective, randomised, multicentre study. Lancet.

[7] Hofman MS, Kasivisvanathan V, Link E (2026). Baseline nodal status on 68Ga-PSMA-11 positron emission tomography/computed tomography in men with intermediate- to high-risk prostate cancer is prognostic for treatment failure: follow-up of the proPSMA trial. Eur Urol Oncol.

[8] Mazzone E, Cannoletta D, Quarta L (2025). A comprehensive systematic review and meta-analysis of the role of prostate-specific membrane antigen positron emission tomography for prostate cancer diagnosis and primary staging before definitive treatment. Eur Urol.

[9] Eiber M, Herrmann K, Calais J (2018). Prostate Cancer Molecular Imaging Standardized Evaluation (PROMISE): proposed miTNM classification for the interpretation of PSMA-ligand PET/CT. J Nucl Med.

[10] Seifert R, Emmett L, Rowe SP (2023). Second version of the Prostate Cancer Molecular Imaging Standardized Evaluation framework including response evaluation for clinical trials (PROMISE V2). Eur Urol.

[11] Rowe SP, Pienta KJ, Pomper MG, Gorin MA (2018). Proposal for a structured reporting system for prostate-specific membrane antigen-targeted PET imaging: PSMA-RADS version 1.0. J Nucl Med.

[12] Ceci F, Oprea-Lager DE, Emmett L (2021). E-PSMA: the EANM standardized reporting guidelines v1.0 for PSMA-PET. Eur J Nucl Med Mol Imaging.

[13] Emmett L, Papa N, Buteau J (2022). The PRIMARY score: using intraprostatic 68Ga-PSMA PET/CT patterns to optimize prostate cancer diagnosis. J Nucl Med.

[14] Shamshirgaran A, Samadi MH, Saeed M (2025). Fifty shades of PSMA-avid rib lesions: a comprehensive review. Cancers (Basel).

[15] Oken MM, Creech RH, Tormey DC, Horton J, Davis TE, McFadden ET, Carbone PP (1982). Toxicity and response criteria of the Eastern Cooperative Oncology Group. Am J Clin Oncol.

[16] Barry MJ, Fowler FJ Jr, O'Leary MP, Bruskewitz RC, Holtgrewe HL, Mebust WK, Cockett AT (1992). The American Urological Association symptom index for benign prostatic hyperplasia. J Urol.

[17] Sweeney CJ, Chen YH, Carducci M (2015). Chemohormonal therapy in metastatic hormone-sensitive prostate cancer. N Engl J Med.

[18] Parker CC, James ND, Brawley CD (2018). Radiotherapy to the primary tumour for newly diagnosed, metastatic prostate cancer (STAMPEDE): a randomised controlled phase 3 trial. Lancet.

[19] Davis ID, Martin AJ, Stockler MR (2019). Enzalutamide with standard first-line therapy in metastatic prostate cancer. N Engl J Med.

[20] Phillips R, Shi WY, Deek M (2020). Outcomes of observation vs stereotactic ablative radiation for oligometastatic prostate cancer: the ORIOLE phase 2 randomized clinical trial. JAMA Oncol.

